# Improving gene expression similarity measurement using pathway-based analytic dimension

**DOI:** 10.1186/1471-2164-10-S3-S15

**Published:** 2009-12-03

**Authors:** Changwon Keum, Jung Hoon Woo, Won Seok Oh, Sue-Nie Park, Kyoung Tai No

**Affiliations:** 1 Bioinformatics & Molecular Design Research Center (BMDRC), Seoul, Korea; 2 Seoul National University Biomedical Informatics (SNUBI), Seoul National University College of Medicine, Seoul, Korea; 3 Dept. of Biotechnology, Yonsei University, Seoul, Korea; 4 Division of Genetic Toxicology, National Institute of Toxicological Research, Korea Food and Drug Administration, Seoul, Korea

## Abstract

**Background:**

Gene expression similarity measuring methods were developed and applied to search rapidly growing public microarray databases. However, current expression similarity measuring methods need to be improved to accurately measure similarity between gene expression profiles from different platforms or different experiments.

**Results:**

We devised new gene expression similarity measuring method based on pathway information. In short, newly devised method measure similarity between gene expression profiles after converting them into pathway based expression profiles. To evaluate pathway based gene expression similarity measuring method, we conducted cell type classification test. Pathway based similarity measuring method shows higher classification accuracy. Especially, pathway based methods outperform at most 50% and 10% over conventional gene expression similarity method when search databases are limited to cross-platform profiles and cross-experiment profiles.

**Conclusion:**

The pathway based gene expression similarity measuring method outperforms commonly used similarity measuring methods. Considering the fact that public microarray database is consist of gene expression profiles of various experiments with various type of platform, pathway based gene expression similarity measuring method could be successfully applied for searching large public microarray databases.

## Background

As microarray experiment has been widely used for various field of biology, public microarray databases have been rapidly growing each year. Currently, the two largest microarray databases, GEO [1] and ArrayExpress [2] are comprised of several hundreds thousands of expression profiles, representing various biological contexts of various species.

In accordance with this expensive collection of large scale gene expression databases, database searching methods have been developed to make the database easily accessible and practically useful. Since the microarray data is deposited in public microarray database as unit of experiment which is consist of several individual gene expression profiles, search methods also have evolved into two way, experiment dataset level search and individual gene expression profile level search.

Most of experiment dataset level search methods are depend on dataset annotation by authors of dataset. Atul B. et al. has been tried to classify the gene expression experiment dataset in GEO (GEO series) by annotating each GEO dataset with medical language terms such as UMLS and SNOMED [3-5] and to make gene expression variation based dataset search possible [6]. Yuelin Z. et al. built GEOmetadb [7], which make text match based GEO dataset search more affordable than original GEO database.

Along the attempts to search large public microarray databases at experiment dataset level, individual gene expression profile level search method has also been conceptualized and developed [8]. GEST [9] is the first implementation of individual profile level search method. It uses Bayesian similarity metric to measure similarity between gene expression profiles. Horton et al. devised fast similarity search algorithm and built web based similar gene expression search system, CellMontage [10,11]. To make cross-platform gene expression profiles search possible, they transformed all gene expression profiles to Unigene ID based gene expression profiles, averaging expression values of genes for corresponding UniGene ID if multiple genes are mapped to a single UniGene ID, then measured similarity between expression profiles using Spearman rank correlation coefficient. Cell type classification as a validation of search power of CellMontage revealed that this method is good enough to search similar expression profiles from the same platform, but not from the different platform [11].

Here we try to improve similar gene expression profile search. For this purpose, we devised a pathway based gene expression similarity measuring method. Our pathway based methods outperform conventional method especially for cross-platform and cross-experiment profile search.

## Methods

### Gene expression data

We used set of gene expression profiles curated by CellMontage group [11]. Each gene expression profile in CellMontage dataset, originally stored in GEO, is manually annotated with cell type and gene expression values of original profiles are averaged to represent expression values of corresponding Unigene ID.

For the classification procedure, we first selected cell types with which at least two different platform types are associated. For each selected cell type, we select at most two gene expression profiles from each platform in the same experiment. After the selection procedure, total 442 gene expression profiles of 40 different cell types from 54 different experiment with 23 different platform types were remained (See Additional file 1). Of these selected gene expression profiles, randomly selected one gene expression profile from each cell type was used as query profile and the other remaining profiles build up search database. Finally, the numbers of gene expression profiles in the query set and the search database are 40 and 402, respectively.

### Pathway data

We used C2 database of MsigDB [12] as pathway data source for pathway summary profiling. Each UniGene ID in gene expression profiles was mapped to corresponding gene symbol of 1,892 pathways in MsigDB using NCBI unigene database [13].

### Pathway expression profiling

Each gene expression profile was converted to pathway centric expression profile by averaging expression values of genes for corresponding pathways. Pathway expression for pathway k, consisted of N genes, is calculated by

where G_i _denotes gene expression for gene i for i = 1, ..., N.

### Gene expression similarity measurement

We used two different scoring methods to measure similarity between gene expression profiles. The first method, conventional method used by Cellmontage, compares common gene set between two comparing gene expression profiles. Let this method call CGSEP(common gene set expression profile) method. Another method compares common pathway set between two comparing pathway expression profiles converted from gene expression profiles. To measure the similarity between gene or pathway expression profiles, we used Spearman rank correlation test. Spearman rank correlation coefficient between profile X and Y is given by

where d_i _= x_i_-y_i_, i = 1, ..., n and x_i_, y_i _= rank of i^th ^gene or pathway in each profile X and Y. Spearman rank correlation coefficient ranges from -1 to 1, where similarity is maximum at 1 and minimum at -1.

### Cell type classification

To evaluate the performance of similarity measuring methods, we conducted cell type classification using nearest neighbor classifier. For each of 40 query gene expression profiles, the similarities to all of 402 gene expression profiles in search database were calculated. Then the profile with highest Spearman rank correlation coefficient was predicted to have the same cell type to query profile. Predicted profile was considered as correct prediction if its cell type is the same as that of query sample. If there is no same cell type profile in search database for a query profile, the search for the given query profile is not counted in classification accuracy assessment. Accuracy of classification is calculated by number of correct predictions divided by number of predictions.

Similar profile search from the profiles of different platform or different experiment is harder than search from the profiles of the same platform or the same experiment [10]. To evaluate the performance of pathway based similarity measuring method, we conducted two more cell type classifications, cross-platform and cross-experiment classification, where search space is consist of profiles whose platforms or experiments are different from that of query.

## Results and discussion

We conducted cell type classification using two different similarity measuring methods and access the performances with overall, cross-platform and cross-experiment search databases.

**Figure 1:**
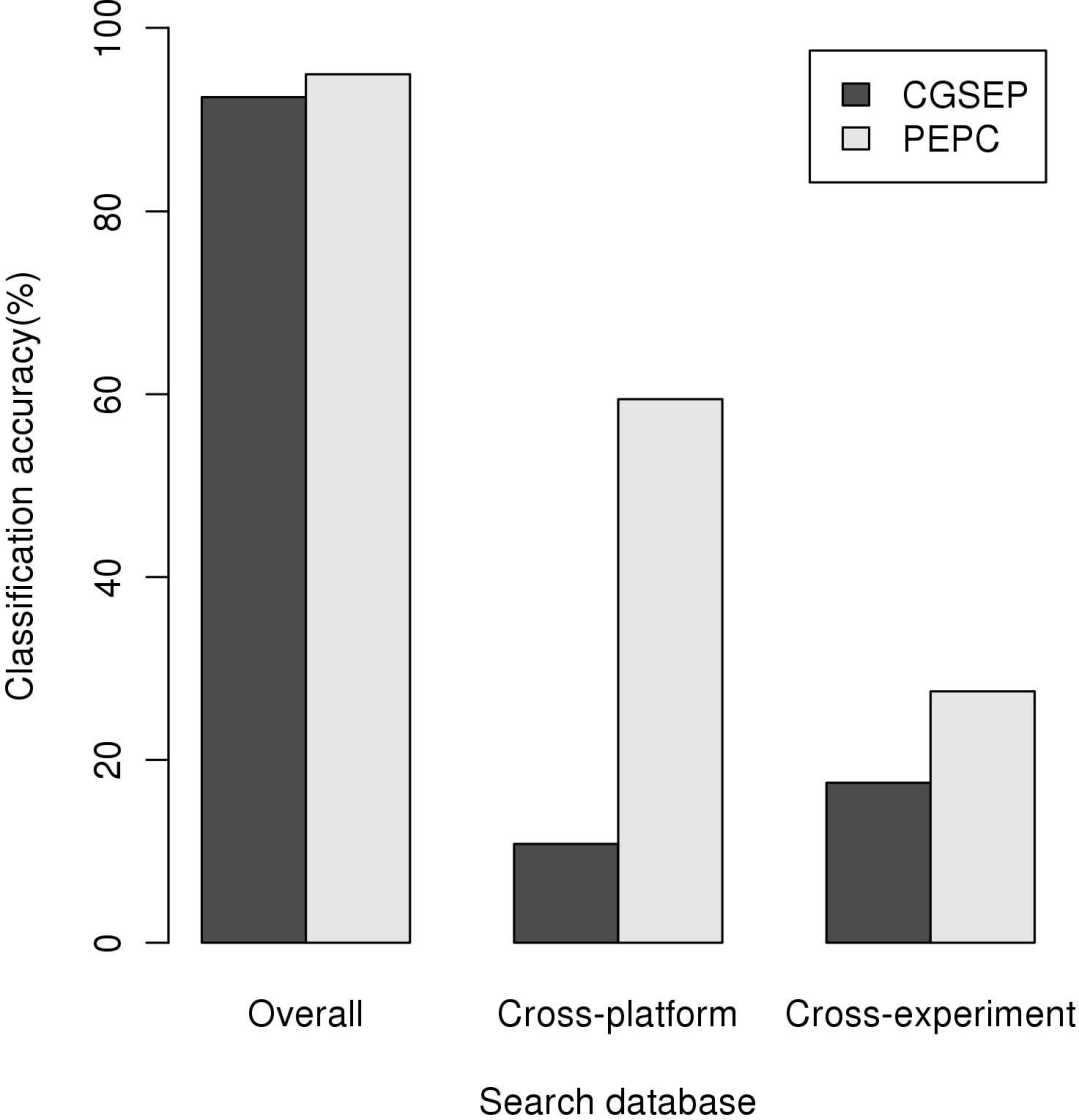
**Cell type classification accuracy**. Cell type classification accuracies using CGSEP and PEPC for three different search databases, overall, cross-platform and cross-experiment.

Barplot shown in figure 1 summarizes all of the classification results. Pathway based similarity measuring method, PEPC, consistently shows higher classification accuracies than CGSEP method for classifications with three different search databases. As an example cases, pathway based method, PEPC, precisely classified cell types of query profile GSM18935 of thalamus cell type with overall search database (Table 1) and query profile GSM12641 of liver cell type with cross-platform search database (Table 2) while CGSEP failed. Pathway based method shows significant improvement when they were applied for cross-platform search database search as PEPC excel CGSEP with 48.6% increased accuracy. Pathway based method also outperformed up to 10% over CGSEP for cross-experiment search, however the improvement is not as significant as the cross-platform classification.

**Table 1:** Top 10 scoring profiles for test profile GSM18935 (thalamus) with overall search database.

Scoring method	Similarity score	Profile ID	Cell type	Platform ID	Study ID
CGSEP	0.62	GSM12688	brain	GPL8300	GSE803
	0.61	GSM12708	brain	GPL8300	GSE803
	0.6	GSM12703	spinal	GPL8300	GSE803
	0.59	GSM12753	spinal	GPL8300	GSE803
	0.58	GSM2885	caudate	GPL91	GSE96
	0.58	GSM2820	cerebral	GPL91	GSE96
	0.58	GSM2886	caudate	GPL91	GSE96
	0.58	GSM2881	thalamus	GPL91	GSE96
	0.58	GSM18443	cardiac	GPL570	GSE1145
	0.57	GSM2897	thalamus	GPL91	GSE96
					
PEPC	0.86	GSM2881	thalamus	GPL91	GSE96
	0.86	GSM2897	thalamus	GPL91	GSE96
	0.84	GSM2885	caudate	GPL91	GSE96
	0.83	GSM2886	caudate	GPL91	GSE96
	0.83	GSM2884	amygdala	GPL91	GSE96
	0.83	GSM2820	cerebral	GPL91	GSE96
	0.83	GSM2828	brain	GPL91	GSE96
	0.82	GSM2874	amygdala	GPL91	GSE96
	0.82	GSM12708	brain	GPL8300	GSE803
	0.82	GSM12688	brain	GPL8300	GSE803

**Table 2:** Top 10 scoring profiles for test profile GSM12641(liver) with cross-platform search database

Scoring method	Similarity score	Profile ID	Cell type	Platform ID	Study ID
CGSEP	0.31	GSM19143	colon	GPL97	GSE1152
	0.3	GSM19142	ileum	GPL97	GSE1152
	0.28	GSM11827	kidney	GPL97	GSE781
	0.25	GSM11810	kidney	GPL97	GSE781
	0.24	GSM4230	skeletal	GPL246	GSE465
	0.24	GSM4231	skeletal	GPL246	GSE465
	0.23	GSM18443	cardiac	GPL570	GSE1145
	0.22	GSM18809	placenta	GPL1074	GSE1133
	0.22	GSM18798	prostate	GPL1074	GSE1133
	0.22	GSM18711	leukocyte	GPL1074	GSE1133
					
PEPC	0.43	GSM2831	liver	GPL91	GSE96
	0.43	GSM12640	liver	GPL92	GSE803
	0.43	GSM2854	trachea	GPL91	GSE96
	0.42	GSM2858	spleen	GPL91	GSE96
	0.42	GSM18949	lung	GPL96	GSE1133
	0.42	GSM18950	lung	GPL96	GSE11750
	0.42	GSM2835	spleen	GPL91	GSE96
	0.42	GSM2844	liver	GPL91	GSE96
	0.41	GSM2834	salivary	GPL91	GSE96
	0.41	GSM12718	liver	GPL8300	GSE803

We next calculated average similarity score of top scoring hit for correct and incorrect classification cases (Table 3). Average similarity score of correct cases is higher than incorrect cases except cross-platform search using CGSEP method, in which CGSEP shows only 10% classification accuracy. Similarity scores for cross-platform search are lower than the other two classifications. This trend is cause by lower expression variations between expression profiles of the same type of platforms than that of different type of platforms [14-16].

**Table 3:** Average similarity scores of top scoring results

Scoring method	Type of top hit	Overall	Cross-platform	Cross-study
CGSEP	Correct	0.91 ± 0.04	0.54 ± 0.12	0.89 ± 0.05
	Incorrect	0.82 ± 0.05	0.54 ± 0.17	0.78 ± 0.11
PEPC	Correct	0.97 ± 0.03	0.74 ± 0.17	0.91 ± 0.06
	Incorrect	0.84 ± 0	0.69 ± 0.18	0.84 ± 0.09

We analyzed further to figure out the reason for low classification accuracy of cross-experiment search. More specifically, our question is why cross-experiment searches show lower classification accuracy than that of cross-platform searches even though the similarity scores for top hits are higher than that of cross-platform searches. To answer this question, we divided cross-experiment search database more specifically into cross-experiment with the same platform profiles and cross-experiment with different platform profiles and conducted cell type classification with those two search databases. Table 4 summarizes the classification accuracy with average similarity scores for correct and incorrect cases for limited search databases. We found again improved classification accuracies with up to 40% higher accuracy compared to original cross-experiment search by pathway based method if cross-experiment search is limited to cross-platform, but this trend is disappeared in search over cross-experiment and the same platform search databases. The average similarity scores to the top hits of same platform top hits are higher than average similarity scores to the top hits of different platforms in cross-experiment search. Even average similarity scores of incorrect cases with the same platform are higher than average similarity scores of correct cases with different platforms. Therefore, the correct profiles of different platforms to the query profile might not score higher than incorrect profiles of the same platforms. This seemed to be the reason for low classification accuracy of cross-experiment search. Considering this reason for low classification accuracy of cross-experiment search, different criteria to evaluate similarity score according to platform type could improve classification accuracy of cross-experiment search.

**Table 4:** Cross-experiment classification results with different search spaces

Scoring Method	Cross-experiment (Same platform)			Cross-experiment (Different platform)		
	**Accuracy (%)**	**Average correct score**	**Average Incorrect score**	**Accuracy (%)**	**Average correct score**	**Average Incorrect score**
CGSEP	17.5	0.89 ± 0.05	0.73 ± 0.24	10	0.54 ± 0.12	0.54 ± 0.17
PEPC	15	0.95 ± 0.06	0.77 ± 0.25	55	0.74 ± 0.17	0.7 ± 0.18

Reduced analytical dimension of pathway expression profiles from gene expression profiles might also contribute improved classification accuracy by pathway based methods. Not all genes in gene expression profile are converted to pathway expression profile for the incompleteness of current pathway information [12,17]. In case of 442 query and profiles in search database used for cell type classification, average 56 ± 15% genes of common gene set for CGSEP method are made up common pathway expression profiles in PEPC. However, the reduced gene expression dimension dose not reduces analytical sensitivity, rather it was reported that classification accuracy is decreased with the addition of feature genes over than the moderate number [18,19]. Likewise, reduced number of genes in the process of pathway expression profiling might increase analytical sensitivity by limiting analytical dimension under moderate size.

We first attempted to use pathway information for gene expression similarity measurement. As previously developed pathway based gene expression analysis methods were successfully improve intact gene expression based analysis methods [20-23], pathway based similarity measuring method outperformed conventional method. Along with the reduced analytical dimension effect described earlier, this improvement seems to be contributed by the averaging effect of expression variation of individual genes caused by both biological and technical reasons. Each human gene do not express or is not detected to expressed constantly even under the same biological condition within a specific microarray platform or across different type of platforms, rather it fluctuates [24,25]. On the other hands, pathway expression, an overall expression pattern of gene set, is robust toward subtle outside stimulation [26]. The pathway based gene expression similarity measuring methods, PEPC, we suggested here, compute pathway level expression by averaging expression of genes mapped to pathway.

Consequently, expression variations of multiple genes are summarized by a robust pathway expression, which represents the activity of the functional unit rather than a component of the unit, thus the pathway based methods result with higher classification accuracy, which demonstrates again that pathway level expression is more robust than individual gene level expression and pathway based similarity scoring methods could be successfully improve similar gene expression profile search.

## Conclusion

We demonstrated that our new gene expression similarity measuring method improved the precision of similar gene expression profile search when it's applied to cell type classification. We showed pathway expression profiling based similarity measuring method outperformed conventional gene expression profile based similarity measuring method over at most 50% for cross-platform profile search and 10% for cross-experiment profile search. At the same time, the classification accuracy shows that the methods still need to be improved, especially for searching similar profiles across different experiment. We believe that our research shed new light on similar gene expression profile search over rapidly growing large microarray databases by showing that integrating gene expression profile with external data such as pathway could improve search accuracy.

## List of abbreviations used

CGSEP: Common Gene Set Expression Profile; PEPA: Pathway Expression Profile for All Gene set; PEPC: Pathway Expression Profile for Common gene set.

## Competing interests

The authors declare that they have no competing interests.

## Authors' contributions

CK designed whole research process, implemented all required methods for the research, analyzed results, drafted and revised the manuscript. JHW devised similarity scoring algorithm with CK and revised the manuscript. WSO and KTN helped the conceptualization of the research process. All authors read and approved the final manuscript.

## Note

Other papers from the meeting have been published as part of *BMC Bioinformatics* Volume 10 Supplement 15, 2009: Eighth International Conference on Bioinformatics (InCoB2009): Bioinformatics, available online at http://www.biomedcentral.com/1471-2105/10?issue=S15.

## Supplementary Material

Additional file 1 **Dataset detail for cell type classification**. All 442 gene expression profiles used for cell type classification are listed with detailed information. Each gene expression profile is annotated with three original GEO accession id, sample id(GSM), experiment id(GSE) and platform id(GPL), and cell type. Click here for file
